# A Retrospective Study on Insertion Torque and Implant Stability Quotient (ISQ) as Stability Parameters for Immediate Loading of Implants in Fresh Extraction Sockets

**DOI:** 10.1155/2019/9720419

**Published:** 2019-11-03

**Authors:** Giuseppe Bavetta, Giorgio Bavetta, Valentina Randazzo, Alessio Cavataio, Carlo Paderni, Vincenzo Grassia, Gianna Dipalma, Ciro Gargiulo Isacco, Antonio Scarano, Danila De Vito, Stefania Cantore, Andrea Ballini, Francesco Inchingolo

**Affiliations:** ^1^Department of Interdisciplinary Medicine, University of Bari “Aldo Moro”, 70121 Bari, Italy; ^2^Private Practice, 90100 Palermo, Italy; ^3^Multidisciplinary Department of Medical-Surgical and Dental Specialties, Second University of Naples, Via Luigi de Crecchio 6, 80138 Naples, Italy; ^4^Department of Medical, Oral and Biotechnological Sciences and CeSi-MeT, University of Chieti-Pescara, 66100 Chieti, Italy; ^5^Department of Basic Medical Sciences, Neurosciences and Sense Organs, University of Bari “Aldo Moro”, 70121 Bari, Italy; ^6^Department of Biosciences, Biotechnology and Biopharmaceutics, University of Bari “Aldo Moro”, 70125 Bari, Italy

## Abstract

**Background:**

To date, insertion torque value (ITV) and implant stability quotient (ISQ) obtained by the Osstell instrument are common clinical methods to assess the initial stability of an implant for a predictable loading procedure. The aim of this current study is to evaluate the ITV and ISQ as stability parameters as part of the decision-making protocol in the adoption of immediate loading in fresh extraction sockets.

**Materials and Methods:**

A total of 41 tapered implants were allocated into two groups: the test group (*n* = 11; 3 males and 8 females; mean age: 62.8 ± 10.7) which received 18 implants as type 1 fresh extraction sockets after teeth removal and the control group (*n* = 7; 4 males and 3 females; mean age: 65.4 ± 9.7) which received 23 implants placed in healed sockets for a period of at least 3 months. Both the ITV and ISQ data were recorded at the time of insertion (*t*_0_). Since ITV (test group) and ITV/ISQ (control group) values were useful for the immediate loading protocol, a screw-retained temporary crown was immediately loaded. ISQ values were recorded after a healing period of 4 months (*t*_1_).

**Results:**

ITV mean values at *t*_0_ in test and control groups were, respectively, 48.61 ± 15.39 and 70.47 ± 14.71, whereas ISQ mean values were 57.55 ± 1.93 and 72.86 ± 5.25, respectively, showing a statistically significant difference (*p* value < 0.001). ISQ mean values at *t*_1_ in either the test or the control group were 68.68 ± 4.20 and 74.54 ± 4.17, not showing a statistical difference. The implant survival rate was 100% in both groups, and no surgical and prosthetic complications were reported during the study.

**Conclusion:**

In conclusion, this study remarked the presence of a residual gap that influenced the ISQ during implant insertion in fresh extraction sockets making this parameter not sufficient for a conclusive decision in the immediate loading, whereas the ITV alone showed to be the best parameter for a final substantial decision.

## 1. Introduction

Implantology is a field of dentistry that has been practiced since many years, thanks to the biological osteointegration principles of Branemark's protocol [1]. The osteointegration, defined as “a direct structural and functional connection between the living bone and the surface of the load-carrying implant,” depends on an atraumatic surgery with the use of surgical motors with speed and torque control, sterile saline solution for irrigation, titanium biocompatibility, and implant primary stability [2].

In 1973, Cameron et al. specified that the micromovements at the bone/implant interface could be tolerated up to a certain threshold between 50 and 150 *μ*m [3]. Therefore, it was common opinion that the micromotions produced by early loading could affect bone healing and induce fibrous tissue encapsulation instead of osteointegration. For this reason, according to the original Branemark's protocol, a no-loaded healing period of 3–6 months following implant placement was essential to achieve adequate implant stability before functional loading [4].

**Figure 1:**
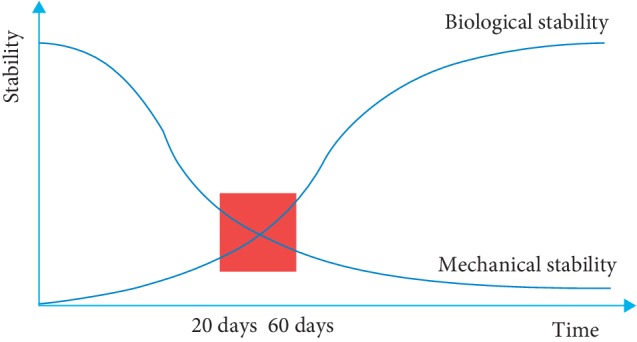
Graphical curve illustrating implant stability as a function of time immediately after placement. Primary implant stability, which is the mechanical stability, decreases in favor of the biological stability that is the osteointegration.

To date, it has been clarified that the measurement of osteointegration can be approached in a quantitative manner, as primary stability and secondary stability are in an inverse relationship [5]. During the postsurgical healing period, between the 20^th^ and 60^th^ days, there is a critical phase due to the peri-implant bone remodeling in which a decay of primary stability occurs in favor of osteointegration (Figure 1); this is a critical time as the implant could be exposed to a higher risk of micromovements, especially in D3 and D4 bone density.

Controlled immediate loading protocols have now been recognized as not interfering with the osteointegration process when applied under well-defined circumstances such as the D1/D2 bone density, in which a decay of primary stability and the insurgence of micromovements are not strong [6–10].

In addition to bone quantity and quality, there are other parameters that may impact the primary implant stability and may play a decisive role for an immediate controlled load; these parameters can be summarized as follows: the implant design (diameter, length, tapered shape, and treated surface) and the surgical technique (underpreparation) [11].

Primary implant stability is a prerequisite for a predictable, long-term secondary stability (osteointegration). Thus, the success of early loading protocols is strictly reliant on the ability and the possibility of the clinician to control the degree of primary implant stability and the evaluation of changes in stability along with healing time.  Table 1 summarizes current available methods for implant stability assessment at pre-, intra-, and postsurgical time points. Though histological and histomorphometric analysis still remains the gold standard in the daily practice, there are strong limitations due to legal and ethical restrictions.

The ITV is expressed in N/cm and is often used to guide loading times [12]. The latest generation of surgical micromotors allows the assessment of the ITV during implant fixture placement.

Nevertheless, the ITV could only be a valid objective parameter used to measure implant stability at the time of insertion unfortunately, as this technique is just a one-time measurement test and inaccurate to evaluate the entire osteointegration process [12].

Alternatively, the resonance-frequency analysis (RFA) has been introduced to provide a noninvasive objective measurement of implant primary stability and to monitor implant stability over the healing period and in the longer term [13]. RFA measures the stiffness and deflection of the implant-bone complex by means of the ISQ scale score. Conventionally, the ISQ scale varies between 40 and 80; the higher the ISQ score, the higher the implant stability. Thanks to this method, the implant can be measured at various intervals in a noninvasive manner, and any changes are recorded prior to the commencement of a restorative therapy. Currently, both the ITV and the ISQ are considered suitable parameters in measuring implant stability and thus early loading protocols [13, 14].

Data from Gallucci and colleagues deduced from the 5^th^ ITI Consensus Conference showed the high predictability of early loading protocols when compared to conventional healing times. However, the same data showed no differences regarding implant survival rates, marginal bone loss, and aesthetic results, and these inferences gave also clinical recommendations for implant loading protocols in case of single implants in partially edentulous patients and fixed prostheses in complete edentulous cases [15]. In the case of immediate loading of single-implant crowns, the recommendations provide an ITV > 20 to 45 N/cm and ISQ > 60 to 65 [16]. In full-arch rehabilitation of totally edentulous patients, an ITV > 30N/cm, ISQ > 60, and minimal implant length > 10 mm have been recommended [17].

Immediate loading protocols have also been used for implants placed into fresh extraction sockets with high predictability in terms of implant survival [18]. Since the bone-implant contact (BIC) is reduced in the fresh extraction socket and limited to the implant apical portion, an adequate surgical protocol for obtaining primary stability is fundamental [19].

Though in this study the initial hypothesis was to confirm the concomitant use of both the ITV and the ISQ as valuable predictors in the immediate loading procedure, the final data obtained showed a different choice. The outcomes demonstrated that the use of ITV alone, when the situation is attested by an ITV score > 30 and an ISQ < 60, could be used as a valuable benchmark to proceed in single-crown immediate loading after fresh extraction sockets, showing that the ISQ based on the scores derived from either group is not determinant in the final decision.

## 2. Results

A total of 18 patients were divided into two groups (Table 2). During the surgical and prosthetic steps of the treatment plan, there were not recorded complications; at *t*_1_, there was no reported implant failure as well as no cases of implant mobility, suppuration, or peri-implant radiolucency in either group.

Eleven patients (*n* = 11; 3 males and 8 females; mean age: 62.8 ± 10.7) in the test group were subjected to 18 implants immediately placed in type 1 fresh extraction sockets, after atraumatic teeth removal. All implants were inserted with an ITV > 30 N/cm (mean = 48.61 ± 15.39) and, thus, were immediately loaded with a screw-retained nonoccluding temporary crown. In this group, for each implant, ISQ values at *t*_0_ were lower than the threshold ISQ values considered sufficient for immediate loading (mean ISQ at *t*_0_ = 57.55 ± 1.93). Within the same group, mean ISQ values recorded after a healing period of 4 months (*t*_1_) (68.66 ± 4.20) showed a statistically significant difference compared to the same values measured at *t*_0_ (*p* value < 0.0005) (see Table 3).

Seven patients (*n* = 7; 4 males and 3 females; mean age: 65.4 ± 9.7) in the control group were treated with 23 implants placed in healed sockets for at least 3 months. In this group, all implants were placed with an ITV > 20 and ISQ > 60 with mean values of 70.47 ± 14.71 and 72.86 ± 5.25, respectively. ISQ values recorded at *t*_1_ showed an average value of 74.54 ± 4.17, with a nonstatistically significant difference with respect to those at *t*_0_ (*p* value = 0.23) (see Table 3).

A comparison of ISQ values from both test and control groups was made. The ISQ values recorded at *t*_0_ and *t*_1_ showed that there was a statistically significant difference between ISQ mean values at *t*_0_ (*Z* score = 3.50; *p* value = 0.00046), but there was no difference in ISQ values between two groups after a healing period of 4 months (*t*_1_) (*Z* score = 2.32; *p* value = 0.0198). Therefore, despite that initial ISQ values at *t*_0_ in the group of fresh extraction socket implants (test group) were significantly lower, after a healing period, they reached similar values as those recorded in the control group, and these values were considered sufficient to evaluate the occurred osteointegration and to move from provisional to final restoration.

## 3. Discussion

Implant primary stability obtained during the implant insertion is essential in achieving osteointegration during the entire healing phase. Immediate implant loading does not result in lack of osteointegration, when the levels of primary stability are not lost during the healing phase.

Therefore, within the whole debate on this topic, and based on the general acceptation, the main issue in this particular field is still a satisfactory prediction of long-term primary stability following an immediate loading procedure in a fresh extraction socket. However, the achievement of this goal as a result of immediate loading protocols depends on few variables that often may be quite elusive, such as the patient's general health condition, the bone quality, the implant outline, the material used, and the surgical skill [20, 21].

Despite the importance of the initial stability of a dental implant to osteointegration, there is still lack of a validated method for a direct and effective predictive measure of the relative movement at the bone-implant interface level [22]. Histomorphometric evaluation of the bone-implant contact (BIC) theoretically could provide information on the implant anchorage, but this approach has been used only in animal studies. ITV and RFA are the techniques most used to assess primary stability. In particular, RFA is a measure of three distinct variables: (1) stiffness of the proper implant, (2) rigidity of the implant-tissue interface, and (3) stiffness of the surrounding bone [13]. The current method of recording RFA is using the Osstell device. It consists of a wireless receptor or SmartPeg, fastened into the implant and triggered by pulse trains emitted from a handheld probe placed in close proximity to the wireless traducer. An algorithm-derived assessment of the resonance frequency recorded results of the ISQ value. Zhou and colleagues together with Scarano and colleagues demonstrated that the BIC was correlated with ISQ values in animals and in retrieved human implants, respectively [23, 24]. An additional study performed by Huang and colleagues investigated the relation between the ISQ and the BIC in an in vitro model study, and a statistically significant correlation was demonstrated between the ISQ and 3D BIC % values measured by micro-CT scanning [25].

This scenario can also be useful, in order to decrease the risk of unwanted fracture, during insertion of a miniscrew for orthodontic applications that need maximum shear bending resistance [26–31].

The combined use of ITV and ISQ parameters to confirm a solid grade of predictability is still a matter of dispute. According to some authors, there is a factual correlation between the ISQ and the ITV, whilst some others were not able to show any statistical correlation [32–36], even if the ITV procedure was conducted with the help of additional devices, a procedure that would have raised the risk of stability and integrity of the area. According to these positions, the weak point of this ITV/ISQ relationship stays on the fact that these two methods are completely independent and incomparable in measuring primary implant stability, suggesting that they should be calculated independently because a high torque does not mean a high ISQ, and vice versa [14].

In this study, for the first time, the ITV and ISQ were evaluated as parameters for implant primary stability in early loading procedures. The outcomes intriguingly showed that initial ISQ values of the test group were significantly lower than those of the control group, with implants placed in the healed extraction site. In the fresh extraction procedure, the implant stability is safely preserved by the contact between the implant surface, the alveolar palatal bone, and 3 to 5 mm of the apical bone over the extraction socket. In this scenario, the surgeon faces a gap between the implant surface and the buccal alveolar bone event similarly seen in the intrabony three-wall vertical defect condition.

A study conducted little more than a decade ago by Turkyilmaz and colleagues demonstrated that the ISQ technique is sensitive to detect marginal bone defects within the implant surface placed in fresh extraction sockets [37]. In a human cadaver study, a linear relationship between the peri-implant vertical bone defect and ISQ values has been demonstrated. In this study, a decay of about 2.97/mm of the ISQ value was shown which corresponded to the information from the manufacturer [37].

**Figure 2:**
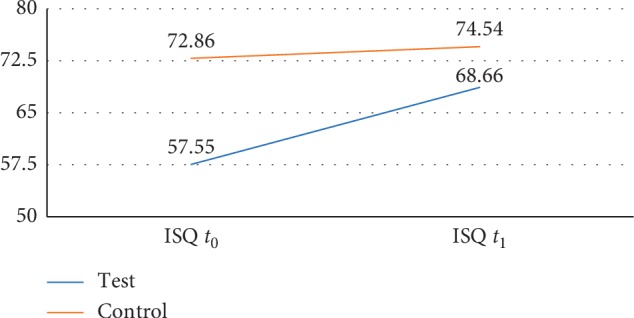
Graph showing ISQ mean value variations in test and control groups at *t*_0_ and *t*_1_. It is worthy of note that, despite that the initial ISQ value variations (*t*_0_) between the two groups are significant, the ISQ values have significantly improved in the test group, finally reaching the average values of ISQ in the control group at *t*_1_. It should be remembered that, in both groups, an immediate loading temporary crown was applied.

In our study, despite that ISQ values at *t*_0_ in the test group were lower than the recommended threshold values (60–65), no immediate loaded implant was lost, and notably, a 100% survival rate was achieved. Of note, there was not statistical difference in ISQ values between the test and control groups after 4 months; in both cases, ISQ outcomes were higher than the commonly accepted threshold for good osteointegration value (ISQ > 65) (see Figure 2).

These data could assume particular interest especially if one considers a different location where the loading procedure was executed for the two groups, either in the upper or in the lower jaw. 11/23 implants were inserted in the mandible in the control group vs the 3/18 implants in the test group. Bearing in mind different bone density between the upper and the lower jaw, practically no difference in terms of ISQ values was seen after consolidated osteointegration.

The registration of ISQ values during the healing period is still a useful method to evaluate the implant healing and the best time to move from temporary to final restoration.

## 4. Materials and Methods

### 4.1. Study Design

This retrospective study was conducted in accordance with the Declaration of Helsinki of 2013 and performed in a private dental practice in Palermo (Studio Bavetta, Piazza Don Bosco, 7H, Palermo, Italy). All subjects gave their informed consent for inclusion before they participated in the study. A total of 18 patients were included in the study, and a total of 41 tapered implants were inserted (Screw-Vent Implant; Zimmer Biomet, USA) between December 2016 and October 2017. Two groups were generated: the test group and the control group (test group = 11 patients and 18 implants and control group = 7 patients and 23 implants).

The inclusion criteria for the test group were as follows:

- At least a type I residual tooth to be replaced in the anterior maxillary or mandibular region, according to the socket classification by Elian et al. [20, 38]
- Absence of acute infection and adequate oral hygiene
- Enough apical bone quantity and residual root to achieve implant primary stability, evaluated by preoperatory CBCT scanning
- Adequate bone density (D1-D2)
- ITV parameter > 30 N/cm
- Vertical dimension occlusion that allows the creation of a nonoccluding temporary crown

The exclusion criteria were as follows:

- American Society of Anesthesiologists (ASA) score ≥ 3
- Type II or III socket, according to the socket classification by Elian et al. [38]
- Presence of active clinical periodontal disease (probing depth ≥ 4 mm and bleeding on probing)
- Presence of acute periapical lesions in the maxillary and mandibular anterior regions
- Smoking history and history of head and neck region radiotherapy

The patients in the control group were recruited among those individuals capable to receive implant placement in the healed socket for at least 3 months, in which ITV and ISQ values were sufficient for the immediate loading protocol (ITV > 20 and ISQ > 60) in accordance with the 5^th^ Consensus Conference as reported by Benic and colleagues [16].

### 4.2. Surgical Procedures

In the test group, tapered implants were immediately placed into fresh extraction sockets after teeth removal (see Table 2). Only one surgeon was in charge to conduct the entire surgical procedures (Giuseppe Bavetta). An atraumatic tooth extraction was performed in local anesthesia by piezosurgery (Piezomed; W&H, Burmoos, Austria), where necessary. The implant was inserted once the integrity of the alveolar socket without a flap procedure was verified according to the guidelines for correct implant placement in the anterior aesthetic zone. For each implant, ITV and ISQ values were recorded at the time of insertion (*t*_0_) by using a surgical micromotor (Implantmed SI-1010; W&H srl, Austria) and an Osstell device (W&H Osstell ISQ module), respectively. The ISQ value was measured with the above-mentioned instrument through magnetic impulses coming from a probe and detected by a device (SmartPeg) screwed on the implant fixture. According to manufacturing instructions, two measurements, at 90° perpendicular to each other, had to be carried out. In all cases, two ISQ measurements for each direction (buccal-palatal and mesiodistal) were performed, and mean values were recorded.

**Figure 3:**
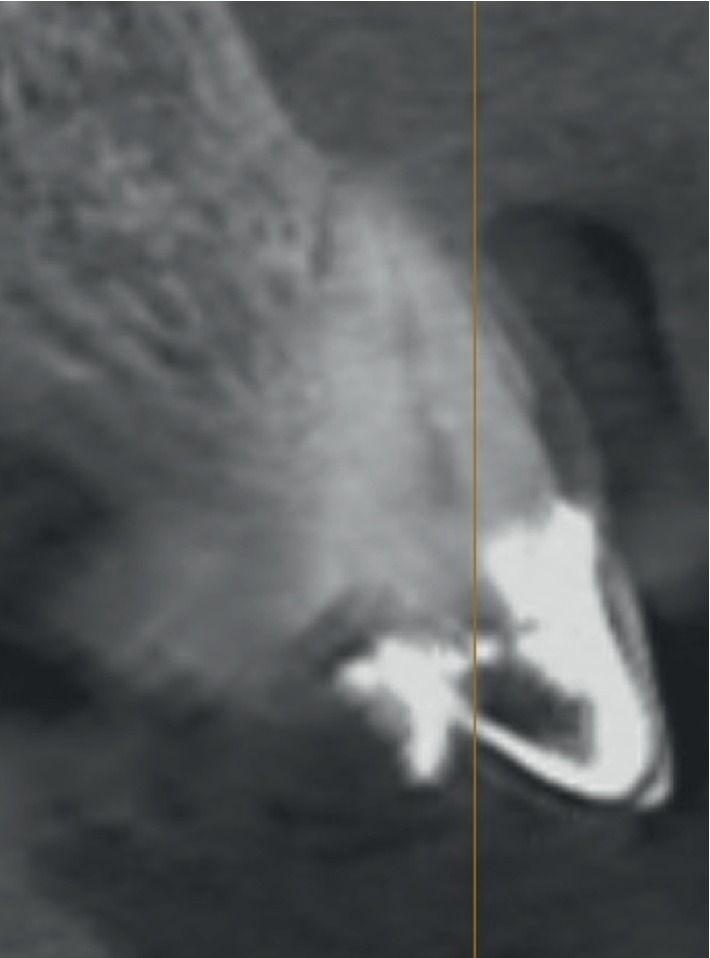
Case 1: initial CBCT for evaluation of the cross section of element 2.1 with a root fracture.

**Figure 4:**
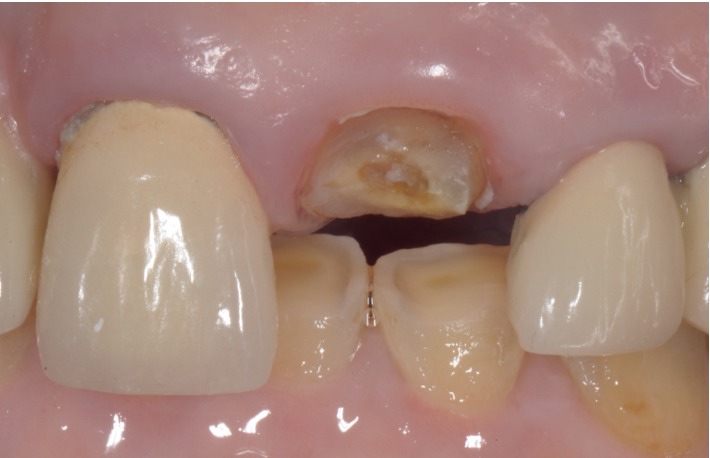
Case 1: front view.

**Figure 5:**
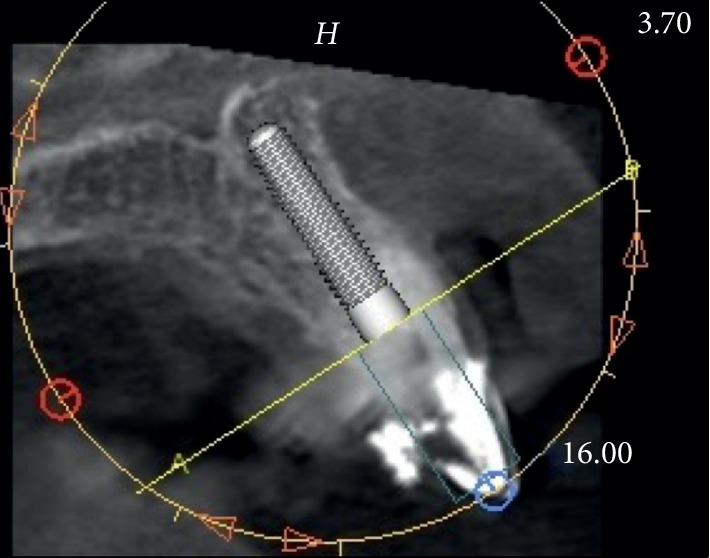
Case 1: virtual ideal implant positioning.

**Figure 6:**
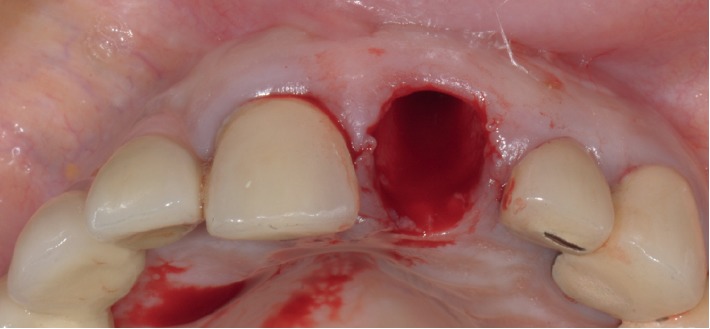
Case 1: fresh extraction socket.

**Figure 7:**
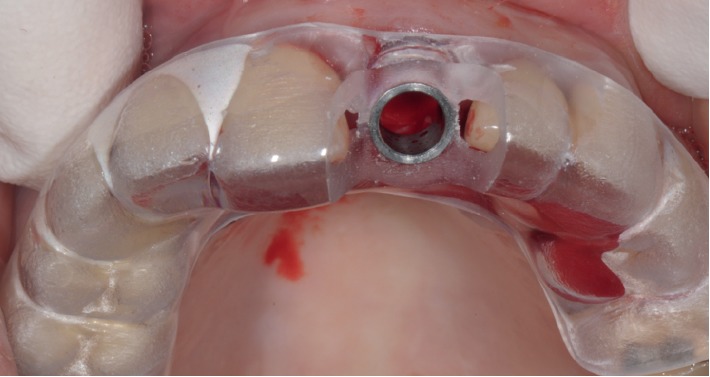
Case 1: template with dental support for guided surgery.

**Figure 8:**
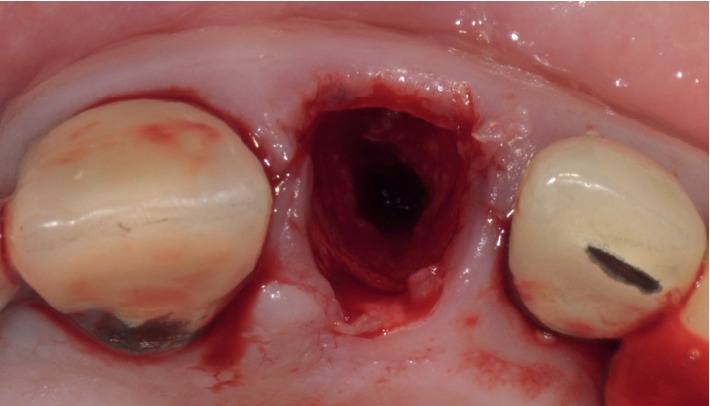
Case 1: implant tunnel.

**Figure 9:**
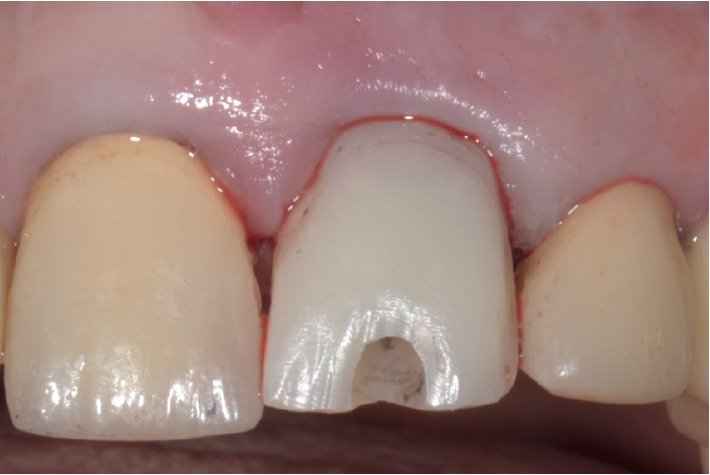
Case 1: immediate screw-retained provisional restoration.

**Figure 10:**
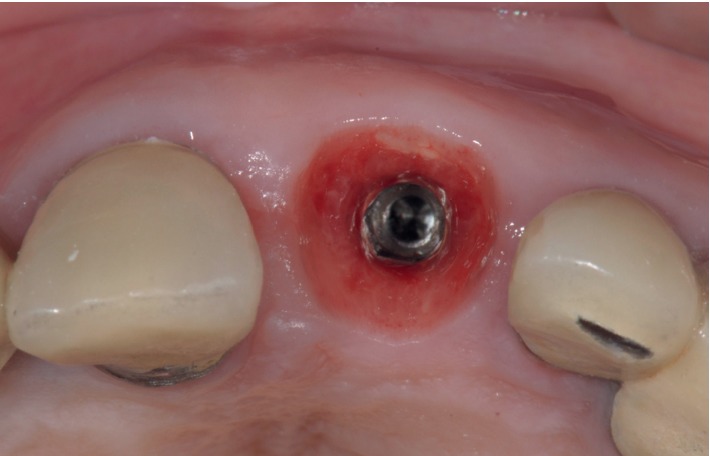
Case 1: buccal contour after 4-month healing.

**Figure 11:**
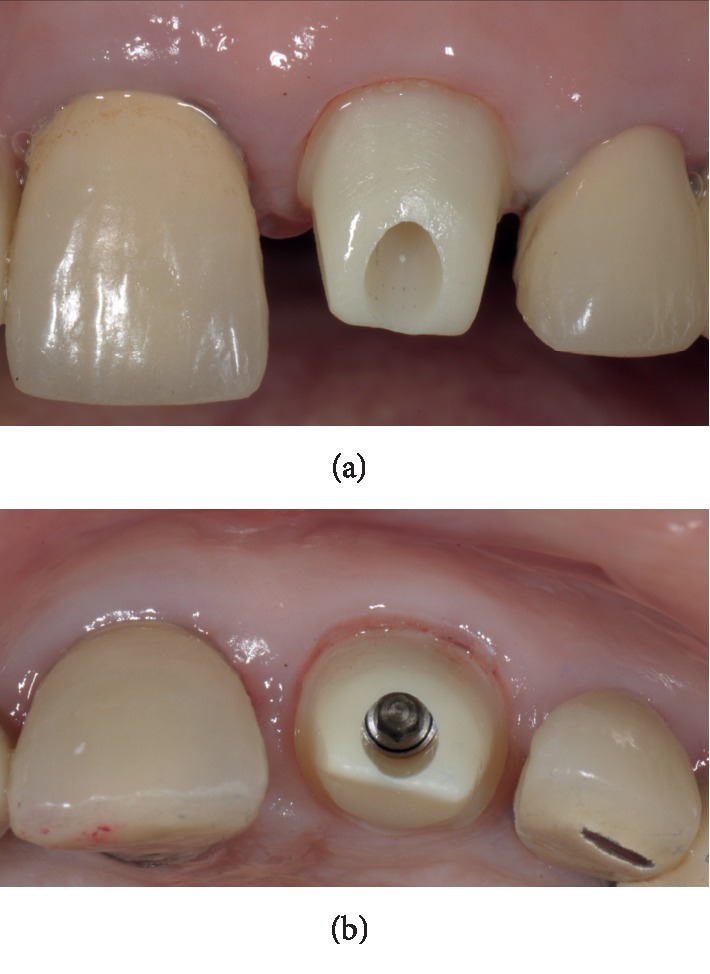
Case 1: custom abutment. (a) Frontal view. (b) Occlusal view.

**Figure 12:**
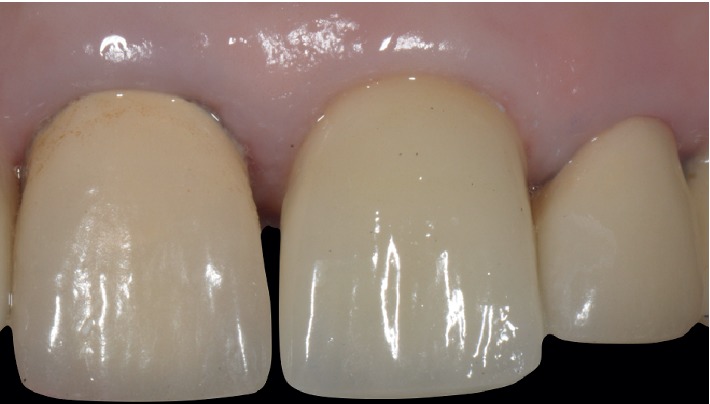
Case 1: final restoration.

**Figure 13:**
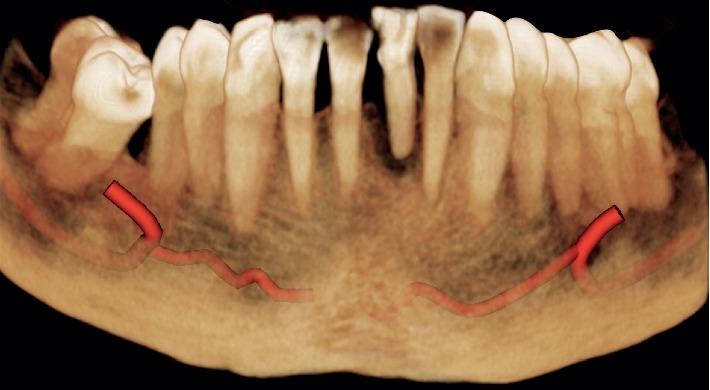
Case 2: front view of 3.1 tooth and initial CBCT for evaluation of large periapical radiolucency with resorption of the root apex; the preservation of the interproximal bone peaks and the reduced mesiodistal diameter of element 3.1 are highlighted.

**Figure 14:**
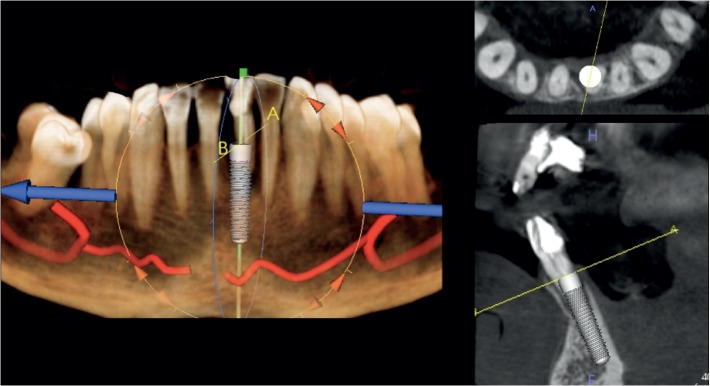
Case 2: virtual ideal implant positioning. An implant design with a diameter of 3.1 mm was used, and the use of the narrow implant allows to respect the minimum safety distances.

**Figure 15:**
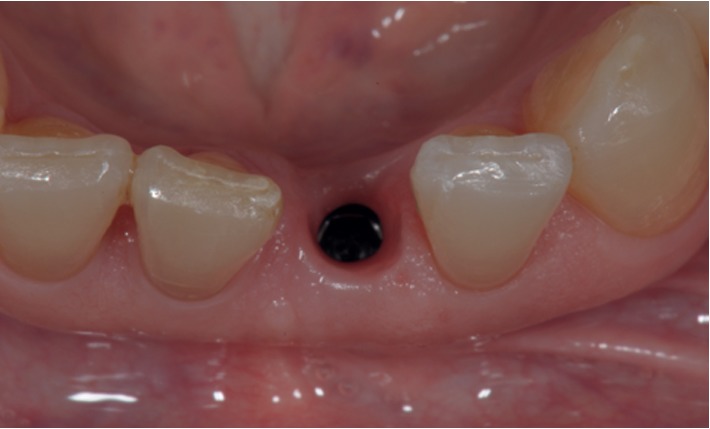
Case 2: buccal contour after 4-month healing.

**Figure 16:**
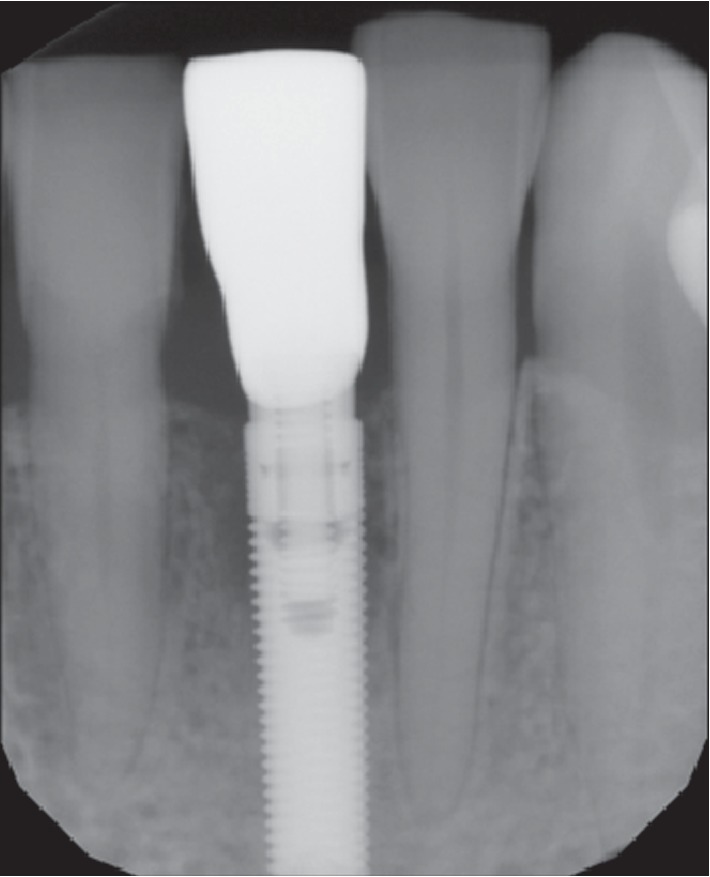
Case 2: radiographic view of final restoration.

Residual gap between the implant surface and the socket buccal plate was filled with the xenograft material, according to the dual zone technique described by Chu et al [39]. Since the ITV parameter was >30 N/cm (inclusion criteria for the test group), a screw-retained temporary crown free from centric and eccentric contact was immediately placed (Figures 3–16). After 4 months, the provisional crowns were removed and ISQ values were assessed again (*t*_1_). Definitive restoration was performed where ISQ values were ≥65. In the control group, tapered implants were placed in healed ridges (at least 3 months) (see Table 2). Since both ITV and ISQ parameters were >20 and >60, respectively, a screw-retained temporary crown was immediately placed. ITV and ISQ parameters at *t*_0_ and *t*_1_ were collected in a similar way to the method carried out for the test group. Finally, definitive restoration was performed when ISQ values were >65.

### 4.3. Statistical Analysis

Descriptive statistics, including the mean values and standard deviations, were calculated for different variables. Comparisons of ITV and ISQ values were made between the control group and the test group. Student's *t*-test was used to compare ISQ mean values recorded at *t*_0_ and *t*_1_ within the same group. The Mann–Whitney *U* test was used to compare different variables between the two groups. A *p* value < 0.001 was considered statistically significant.

The GraphPad InStat statistical software (GraphPad Software, San Diego, CA; available from http://www.graphpad.com) was used for analysis.

## 5. Conclusion

Although current protocols strongly suggest the use of ITV, ISQ, and RFA methodologies, in order to make a solid decision towards the immediate loading procedure, this study showed that the ITV alone could be enough in the decision-making process. The outcomes, though still low in number, clearly showed that ISQ values recorded at the time of implant placement are not sufficient as a conclusive parameter for an immediate loading protocol after extraction socket since the ISQ may give either incorrect values or incongruence data because of the presence of a residual gap in extraction sockets. In this viewpoint, the ITV alone could be considered a better parameter in predicting the success of primary stability of implants inserted after fresh extraction, indicating whether to use an immediate loading procedure.

However, ISQ monitoring is a useful method to evaluate the implant healing and to choose the best time to move from the provisional to the final restoration. To conclude, we are well aware that further research, cases, and analysis are needed to validate and confirm our position regarding the validity of the ITV alone in the execution of immediate loading procedures, especially in regard to particular situations such as poor bone quality and quantity and multiple implants or augmentation dealings.

## Figures and Tables

**Table 1:** Current available methods for implant stability assessment at pre-, intra-, and postsurgical time points; for each method, advantages and disadvantages have been reported.

	Method	Evaluation	Presurgery	Intrasurgery	Postsurgery	Advantages	Disadvantages	Objectivity
*Noninvasive methods*								
Percussion test	Percussion with tool handle	Qualitative: resonance of the implant in the bone, clear sound, gloomy sound	Not possible	Certain reliability	Certain reliability	Simple and not expensive	Subjective, poor sensitivity	Doubtful reliability
Radiographic analysis	Endoral RX	Quantitative and qualitative: radiating transparency along the bone implant surface and marginal bone level	Certain reliability	Certain reliability	Certain reliability	Simple and not expensive	Two-dimensional examination, not standardizable, not for short follow-ups (<6 weeks)	Not evaluable
Periotest	Electronic pulse sequence	Quantitative. damping of the periodontium and tooth mobility	Certain reliability	Certain reliability	Certain reliability		Subjective, poor sensitivity, values are not significant	Certain reliability, but more information is needed
Measurement of shear strength (Osseo-Care)	Surgical, for example, by means of a tap	Quantitative: cut resistance of the implant site and bone density	Certain reliability	Highest reliability	Certain reliability		Limited to surgery	Certain reliability
Reverse torque test	Reverse torque test of 20 N/cm of the exposed implant	Quantitative: unscrewing the implant	Not possible	Not possible	Certain reliability		Bone deformation, provocation of failures, false positives on implants longer than 13 mm	Certain reliability
RFA	Magnetic pulses picked up by SmartPeg	Quantitative and qualitative: evaluation of the degree of bone-implant contact on a scale from 1 to 100	Not possible	Highest reliability	Highest reliability	Evaluation of immediate loading and evaluation of the increase in the bone-implant contact for the purpose of final prosthetics		Certain reliability, but more information is needed

*Invasive methods*								
Histologic analysis	Sampling using a milling technique	Bone quantity and bone quality (histomorphometry)	Doubtful reliability	Doubtful reliability	Doubtful reliability	High quality	Invasive	Highest reliability
Removal torque measurement	Disarming test, manual/electronic force application on the implant	Quantitative: force necessary to separate bone-implant unit	Not possible	Doubtful reliability	Certain reliability		Invasive, depends on the implant geometry	Certain reliability

**Table 2:** Study design: all data and variables of test and control groups are summarized.

Patient ID	Implant ID	Sex	Age	Implant Position	Implant (diameter × length) (mm)	ITV (N/cm)	Mean ISQ at *t*_0_	Mean ISQ at *t*_1_
*Test group*								
1	1	F	55	3.1	3.1 × 16	34	58.5	66.5
	2			4.1	3.1 × 16	50	59	67
2	3	F	58	3.1	3.1 × 16	41	56	65.5
3	4	F	59	1.4	3.7 × 16	80	57	68.5
4	5	M	55	1.1	3.7 × 16	51	59.5	67
5	6	F	73	1.4	3.7 × 16	45	56	65.5
6	7	F	43	1.4	3.7 × 16	51	59.5	69
7	8	F	77	1.4	3.7 × 16	36	55.5	66.5
	9			1.5	4.1 × 11.5	67	59.5	65.5
8	10	M	61	1.4	3.7 × 16	80	59.5	63
9	11	F	61	1.1	3.7 × 16	32	54.5	69.5
	12			1.2	3.7 × 13	43	56	62
	13			1.3	3.7 × 16	37	56	69
	14			1.4	3.7 × 13	32	59.5	74
10	15	M	74	1.3	3.7 × 16	48	56.5	71
	16			1.4	3.7 × 16	54	59.5	76.5
	17			1.5	3.7 × 13	63	59.5	74
11	18	F	75	2.1	3.7 × 16	31	54.5	76
			*μ* = 62.8			*μ* = 48.61	*μ* = 57.55	*μ* = 68.66
			*σ* = 10.7			*σ* = 15.39	*σ* = 1.93	*σ* = 4.20

*Control group*								
12	19	M	69	3.4	3.7 × 11.5	52	68.5	73.5
13	20	M	79	4.4	3.7 × 10	80	78	80
	21			4.5	3.7 × 10	65	83.5	84
14	22	F	64	1.5	3.7 × 13	80	73	77.5
	23			1.4	3.7 × 16	80	77	77
	24			1.3	3.7 × 16	80	74	77
	25			1.1	3.7 × 16	70	80	73.5
	26			2.1	3.7 × 16	80	81	69.5
15	27	M	70	4.5	3.7 × 8	80	81	82
	28			4.6	3.7 × 8	80	72	74
	29			3.5	3.7 × 8	35	67	68.5
	30			3.6	3.7 × 8	35	66	68
16	31	F	47	1.2	3.7 × 13	62	67	69
17	32	F	63	4.4	3.7 × 16	80	76.5	77.5
	33			4.2	3.7 × 16	80	70.5	74.5
	34			3.2	3.7 × 16	80	74.5	75.5
	35			3.4	3.7 × 16	80	68	70.5
18	36	M	66	1.5	3.7 × 13	80	68.5	75
	37			1.4	3.7 × 13	80	72	73
	38			1.3	3.7 × 13	62	72.5	71
	39			2.3	3.7 × 13	49	66.5	73
	40			2.4	3.7 × 13	71	68.5	77
	41			2.5	3.7 × 13	80	70.5	74
			*μ* = 65.4			*μ* = 70.47	*μ* = 72.86	*Μ* = 74.54
			*σ* = 9.7			*σ* = 14.71	*σ* = 5.25	*σ* = 4.17

**Table 3:** Statistical analysis of mean ITV and ISQ values recorded at *t*_0_ and *t*_1_ in both test and control groups.

Comparison	Statistical tests	Results	Conclusions^*∗*^
ISQ *t*_0_ (test group) vs. ISQ *t*_1_ (test group)	Student's *t*-test	*t* = 10.19; *p* value < 0.0001	Extremely significant
ISQ *t*_0_ (control group) vs. ISQ *t*_1_ (control group)	Student's *t*-test	*t* = 1.20; *p* value = 0.23	Not significant
ISQ *t*_0_ (test group) vs. ISQ *t*_0_ (control group)	Mann–Whitney *U* test	*Z* score = 3.50; *p* value = 0.00046	Significant
ISQ *t*_1_ (test group) vs. ISQ *t*_1_ (control group)	Mann–Whitney *U* test	*Z* score = 2.32; *p* value = 0.0198	Not significant
ITV (test group) vs. ITV (control group)	Mann–Whitney *U* test	*Z* score = 3.58; *p* value = 0.00034	Significant

^*∗*^ *p* value < 0.001 was considered statistically significant.

## Data Availability

All experimental data used to support the findings of this study are available from the corresponding author upon request. The authors have annotated the entire data building process and empirical techniques presented in this paper. The data underlying this article are not freely available by agreement with partners to protect their confidentiality.
